# Epigenetic modulation of antitumor immunity for improved cancer immunotherapy

**DOI:** 10.1186/s12943-021-01464-x

**Published:** 2021-12-20

**Authors:** Enyong Dai, Zhi Zhu, Shudipto Wahed, Zhaoxia Qu, Walter J. Storkus, Zong Sheng Guo

**Affiliations:** 1 Department of Oncology and Hematology, China-Japan Union Hospital of Jilin University, Changchun, Jilin China; 2 UPMC Hillman Cancer Center, Pittsburgh, PA USA; 3 Department of Surgery, University of Pittsburgh School of Medicine, Pittsburgh, PA USA; 4 Department of Surgical Oncology, China Medical University, Shenyang, China; 5 Department of Immunobiology, Yale School of Medicine, New Haven, CT USA; 6 Department of Microbiology and Molecular Genetics, University of Pittsburgh School of Medicine, Pittsburgh, PA USA; 7 Departments of Dermatology, Immunology, Pathology and Bioengineering, University of Pittsburgh School of Medicine, Pittsburgh, PA USA; 8 Department of Immunology, Roswell Park Cancer Institute, Buffalo, NY USA

**Keywords:** DNA methylation, Histone modifications, Epigenetic reprogramming, Metabolic reprogramming, Heterogeneity, Immune cells, T cells, Antitumor immunity

## Abstract

Epigenetic mechanisms play vital roles not only in cancer initiation and progression, but also in the activation, differentiation and effector function(s) of immune cells. In this review, we summarize current literature related to epigenomic dynamics in immune cells impacting immune cell fate and functionality, and the immunogenicity of cancer cells. Some important immune-associated genes, such as granzyme B, IFN-γ, IL-2, IL-12, FoxP3 and STING, are regulated via epigenetic mechanisms in immune or/and cancer cells, as are immune checkpoint molecules (PD-1, CTLA-4, TIM-3, LAG-3, TIGIT) expressed by immune cells and tumor-associated stromal cells. Thus, therapeutic strategies implementing epigenetic modulating drugs are expected to significantly impact the tumor microenvironment (TME) by promoting transcriptional and metabolic reprogramming in local immune cell populations, resulting in inhibition of immunosuppressive cells (MDSCs and Treg) and the activation of anti-tumor T effector cells, professional antigen presenting cells (APC), as well as cancer cells which can serve as non-professional APC. In the latter instance, epigenetic modulating agents may coordinately promote tumor immunogenicity by inducing de novo expression of transcriptionally repressed tumor-associated antigens, increasing expression of neoantigens and MHC processing/presentation machinery, and activating tumor immunogenic cell death (ICD). ICD provides a rich source of immunogens for anti-tumor T cell cross-priming and sensitizing cancer cells to interventional immunotherapy. In this way, epigenetic modulators may be envisioned as effective components in combination immunotherapy approaches capable of mediating superior therapeutic efficacy.

## Introduction

Both genetic and epigenetic changes are essential contributors to the onset of carcinogenesis, tumor progression and metastasis [1, 2]. In a broad sense, the incidence of cancer is directly related to biologic/genetic age. DNA methylation, a prominent epigenetic regulation mechanism, varies over a lifetime and functions as an important component of the “epigenetic aging” process. Notably, recent studies have confirmed that epigenetic aging plays a major role in tumorigenesis [3, 4].

Epigenetic alterations contribute to carcinogenesis by impacting multiple oncogenic vs. tumor suppressor gene pathways in a broad range of tissue histologies [5, 6], as well as by impacting the activation, differentiation, and functional fate of immune cells such as T cells and NK cells that serve as a surveillance mechanism against cancer [4, 7–10]. Some epigenetic changes occur early in development, preceding the onset of tumor development [11–13]. Indeed, a recent study showed that tissue environment-induced epigenetic programming initiates tumorigenesis [14], with Feinberg and others proposing an epigenetic progenitor origin for human cancer [15, 16]. These findings provide a strong rationale for the use of epigenetic drugs not only as cancer therapeutics, but also for the prevention of cancer where they may coordinately target “normal” cells including immune cells and precancerous cells.

Epigenetic-based therapy aims to modulate transcriptional programming affecting various signaling pathways in immune cells, other normal cells and/or cancer cells, thus affecting the fate of each of these cell populations [17–19]. Thus, *epigenetic drugs* are chemicals that act on the epigenome of cells to exert their functions. These drugs include inhibitors of DNA methyltransferases (DNMTs), DNA demethylases, histone deacetylases (HDACs), histone acetyltransferases (HATs), histone methyltransferases (HMTs), histone demethylases (HDMs) and other relevant enzymes. In addition, microRNAs (miRNAs) and long non-coding RNAs (lncRNAs) are also important epigenetic mediators for a variety of key biological processes, including carcinogenesis and the immune response, two pivotal targets in effective cancer therapy [20, 21]. However, due to space limitations, these classes of molecules will not be discussed in detail in this report, with readers instead referred to several outstanding reviews focused on the role of miRNAs/lncRNAs in the cancer setting [20, 21].

In this review, we summarize current understanding of epigenetic changes in the immune cells during normal development, cancer progression and on-treatment with cancer therapeutic agents. We discuss potential innovative strategies to target immune cells as well as cancer cells using epigenetic modulators for the development of more effective targets in combination immunotherapies.

## Overview of epigenetic pathways, the enzymes and inhibitors

### DNA methylation

DNA methylation, as one of the major epigenetic marks, can be mitotically inherited and is involved in stabilizing repression of gene transcription, especially when it is located close to the transcription start sites of mammalian genes [22]. The best-studied covalent modification on DNA is 5-methylcytosine (5mC), a mark catalyzed by DNA methyltransferase (DNMT). In mammalian genomes, 5mC exists mostly in the CpG dinucleotide context with 70–80% of CpGs being methylated among the 28 million CpG dinucleotides in the human genome [23]. The DNMT family of enzymes catalyze the methylation using S-adenosyl methionine (SAM) as the methyl donor. Based on substrate specificity, m5C methyltransferases are found in animals beginning with the echinoderms, while m6A and m4C methyltransferases are found primarily in prokaryotes. In mammals, three active DNMTs are found and designated as DNMT1, DNMT3a, and DNMT3b. As there are many excellent reviews on these enzymes and DNA methylation, we refer our readers to a few of these comprehensive articles [23–25].

DNA demethylation occurs either by passive or active processes. 5-Hydroxymethylcytosine is a key nexus in demethylation that can either be passively depleted through DNA replication, or actively converted to cytosine through iterative oxidation and thymine DNA glycosylase (TDG)-mediated base excision repair. In the active demethylation pathway, ten-eleven translocation (TET) family enzymes and TDG are involved [26]. TET family dioxygenases and DNA demethylation exert pleiotropic biologic effects on stem cells and cancer cells [27], and play important roles in immune cell development [28]. TET proteins catalyze oxidization of 5-methylcytosine to 5-hydroxymethylcytosine and further oxidation products in DNA. Oxidized methylcytosines facilitate DNA demethylation and represent novel epigenetic marks. TET loss-of-function is strongly associated with cancer, with TET2 loss-of-function mutations frequently observed in hematological malignancies that are resistant to conventional therapies [29, 30]. Importantly, TET proteins govern cell fate decisions during the development of various cell types by activating cell-specific gene programming [29–31]. Two studies in 2015 showed that TET1 serves as a suppressor of hematopoietic malignancies [32, 33]. Later, Cimmino et al. showed that restoration of TET2 function blocks aberrant self-renewal in leukemia cells, thereby blunting disease progression [34]. Interestingly, the loss of TET2 expression promotes CD8^+^ T memory cell differentiation [35]. Perhaps more importantly, disruption of TET2 expression enhances the therapeutic efficacy of adoptively-transferred CD19-targeted CAR-T cells in the setting of hematologic malignancies [36]. These results have significant implications in cancer immunotherapy. In this context, we will discuss the role of TET in the maintenance of Treg cells later in this article.

### Histone acetylation

**Fig. 1:**
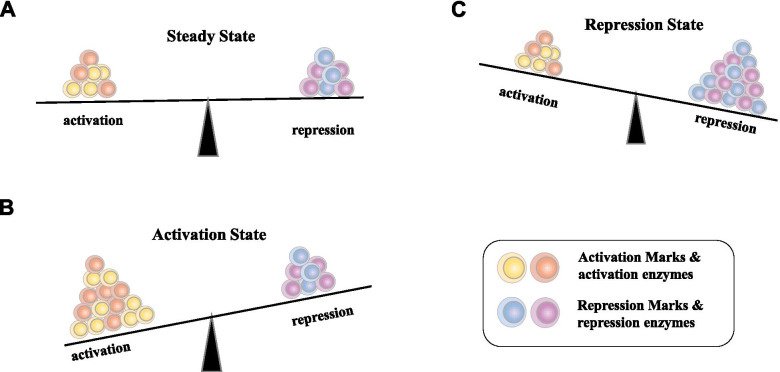
Overview of balanced states of transcription status maintained by the versatile chromatin proteins and histone posttranslational modifications, as well as DNA methylation in the promoter region. The histone-modifying enzymes can be divided into two classes for activation and repression. The chromatin states are maintained and balanced by a number of activation marks and repression marks. Histone marks highlighted in bold represent hallmarks of euchromatin (H4K16ac) and heterochromatin (H3K9me3 and H3K27me3), respectively. DNA methylation and histone modifications on the promoter region cross-talks [44], to dictate the transcriptional activity of the gene. The repressive marks may include H3K9me3, H3K27me3, H4K20me2/3, H2AK119ub, H3R2me, biotinylation, sumoylation and citrullination, while activation markers may include H3K4me1/2/3, H3K9me1, H3K27me1, H4k20me1, H3K36me1/2/3, H3K79me1/2/3, H3K27ac and butyrylation [45]

Histones can undergo multiple forms of posttranslational modifications (PTMs) including acetylation, methylation, phosphorylation, ubiquitination, as well as ADP-ribosylation, SUMOylation and citrullation [37]. These make up the so-called “histone code” that regulates chromatin structure, the recruitment of remodeling enzymes and the modulation of gene activities. The enzymes regulating epigenetic modifications on histones are classified as writers, erasers, readers, or movers based on their effects [38–40] (Fig. 1). Acetylation of histone lysine residues affects genome organization and function. The acetylation of histone H3 and H4 are dictated by two sets of enzymes: histone acetyltransferases (HATs) and histone deacetylases (HDACs). These two sets of enzymes determine the acetylation status of histones and work in a reversible manner. HATs are responsible for bringing about the targeted acetylation of histones and other factors [41–43]. Based on their subcellular localization, HATs are historically divided into two classes. Type A are those located in the nucleus, where they are involved in the transcriptional regulation of genes through acetylation of nucleosomal histones in the chromatin. One conserved feature of HATs is that they contain a bromodomain, which helps in recognizing and binding to the acetylated lysine residues on histone substrates. Gcn5, p300/CBP, and TAFII250 are type A HATs. Class B are HATs localized to the cellular cytoplasm. They function to acetylate newly synthesized histones prior to their assembly into nucleosomes. A key feature of Class B HATs is that they lack a bromodomain, as their targets are unacetylated, in contrast to class A HATs. Hat1 is one of the few known examples of a type B HAT. More recently, based on sequence homology as well as shared structural features and functional roles, HATs have been grouped into several different subfamilies, including, Gcn5-related N-acetyltransferases (GNATs), MYST HATs, and others.

In humans, there are 18 HDAC enzymes divided into four classes: Class I for Rpd3-like proteins (HDAC1, HDAC2, HDAC3, and HDAC8); Class II for Hda1-like proteins (HDAC4, HDAC5, HDAC6, HDAC7, HDAC9, and HDAC10); Class III for Sir2-like proteins (SIRT1, SIRT2, SIRT3, SIRT4, SIRT5, SIRT6, and SIRT7); and finally, class IV protein (HDAC11) [46]. Interestingly, Class I HDACs are expressed ubiquitously, whereas the expression of class II HDACs is more restricted [1, 47, 48]. These enzymes and their functions in cancer have been extensively reviewed [49, 50].

We will now provide a short overview of these enzymes and discuss newly discovered functions associated with these bioactive molecules on immune cells. Of the class I enzymes, HDAC-1, − 2, − 3 catalytic activities rely on their individual co-repressor complexes. They can be localized in cytoplasm and nucleus, with some members also found on the plasma membrane (HDAC3, − 8). HDAC-1/2 double knockout affects CD4^+^ T cell lineage differentiation in part by downregulating TCR signaling, leading to oncogenic transformation in immature T cells [51, 52]. HDAC3 is an epigenetic inhibitor of cytotoxic programming in CD8^+^ T cells [53]. All class IIa HDACs contain an extended N-terminal domain with conserved serine residues and other motifs impacting subcellular localization and function [54]. These serine residues are targets for phosphorylation by kinases, regulating HDACs nuclear export. All class IIa HDACs contain nuclear localization signal sequences. Of the class IIb HDACs, HDAC6 is a unique member that not only participates in histone acetylation and deacetylation, but also targets several non-histone substrates, such as α-tubulin, cortactin, and heat shock protein 90 (HSP90) to regulate cell proliferation, invasion and metastasis within the TME [55]. For example, HDAC6 plays a non-canonical role in the regulation of anti-tumor immune responses, as well as tumor invasion/dissemination in the setting of breast cancer [56]. HDAC10 is another important member regulating immune cell functions. HDAC10 deletion promotes enhanced Foxp3^+^ Treg cell suppressor function [57]. All 7 NAD^+^-dependent class III HDACs, or SIRTs, have been identified in the cytoplasm, nucleus and mitochondria. SIRT proteins play an important role in the survival and drug resistance of tumor cells. Furthermore, SIRT1 limits the function and fate of MDSCs in tumors by orchestrating HIF-1α-dependent glycolysis [58]. SIRT inhibitors induce cell death and p53 acetylation through targeting both SIRT1 and SIRT2 [59]. As the newest member of the family and the only class IV member, HDAC11 is a regulator of diverse immune functions [60]. For example, HDAC11 plays a physiologic role as a multifaceted regulator of neutrophils [61]. HDAC11 targeting enhances Foxp3^+^ Treg function, and T cells lacking HDAC11 mediate increased effector functions [62, 63]. Interestingly, HDAC11 regulates type I IFN signaling through defatty-acylation of SHMT2 on lysines, rather than its conventional role as a histone deacetylase [64].

Li et al. have recently provided an extensive review on these enzymes, including their sub-cellular distribution, biological functions, respective inhibitors, their underlying biological mechanisms of action and the potential translation of these findings for cancer therapy [50]. It is important to note that HDACs possess many diverse biological functions. These include, but are not limited to, transcriptional regulation, metabolism, hypoxia and angiogenesis, redox and oxidative stress, DNA damage response, cell cycle, cell apoptosis, modulation of degradation system, epithelial-mesenchymal transition, cancer stem cell status and cellular fate/senescence. Of note, HDACs exert these functions not only through epigenetic mechanisms, but also via their action on actionable proteins other than histones.

The bromodomain and extra-terminal domain (BET) family includes BRD2, BRD3, and BRD4 and the testis-restricted BRDT. They function as epigenetic readers by binding to specific acetylated lysine residues on histone tails, resulting in facilitated assembly of transcription complexes including transcription factors and transcriptional machinery like RNA polymerase II. Many inhibitors for BET proteins are being developed and some are in clinical trials for cancer treatments [65].

### Histone methylation

Histone methylation is the third major type of epigenetic modification [45]. To complete this reversible process, two major classes of enzymes that catalyze the addition of a methyl group (i.e., histone methyltransferases [HMTs]) are involved. HMTs that methylate arginine residues, are called protein arginine methyltransferases (PRMTs) [66]; and those that methylate lysine residues, histone lysine methyltransferases (HKMTs) [67]. Key lysine and arginine methyltransferases in the cancer setting include EZH2, G9a, disruptor of telomeric silencing 1-like protein (DOT1L), and PRMTs 1 and 5 [39]. Another set of enzymes called histone demethylases oppose this process [68]. Hypoxia induces the rapid and HIF–independent induction of histone methylation in a range of human cultured cells, which reprograms chromatin [69]. There has been significant interest in developing targeted small molecule inhibitors against these HMTs and histone demethylases (KDMs) [70].

Some HMTs and KDMs play fundamental roles in immune cell activation, differentiation and functional stability. We now highlight several recent studies to illustrate these points. The HMT Setd2 is indispensable for V(D)J recombination during early T or B cell development [71]. A second HMT SETDB1 controls T helper cell lineage integrity by repressing endogenous retroviruses [72]. A third HMT DOT1L is essential for humoral (i.e., B cell-mediated) immune responses [73].

Lysine-specific demethylase 1 (Lsd1/KDM1a) demethylates histone H3 on Lys4 or Lys9 (H3K4/K9) and constitutes an indispensible epigenetic regulator of hematopoietic cell differentiation [74]. Shi et al. demonstrated that inhibition of LSD1 in cancer cells increases repetitive element expression, including endogenous retroviral elements (ERVs), and decreases expression of RNA-induced silencing complex (RISC) components. This leads to double-stranded RNA (dsRNA) stress and activation of type 1 interferon production, leading to the stimulation of anti-tumor T cells and restricted tumor growth. Since LSD1 functions as a potent inhibitor of anti-tumor immunity [75], it is not surprising that LSD1 inhibitors promote anti-tumor immunity and improve the therapeutic efficacy of immune checkpoint blockade in various tumor models [75, 76].

### Histone phosphorylation

Phosphorylation is another form of histone PTM which is highly dynamic. It takes place on serine, threonine and tyrosine residues, and predominantly happens in the histone N-terminal tail, being controlled by the competing actions of kinases and phosphatases [77, 78]. As an essential part of the ‘histone code’ [79], this PTM plays a key role in DNA damage repair, chromatin compaction, transcriptional regulation, and a range of biological processes including oncogenesis and cancer progression [78].

Recent studies have illustrated the important roles of histone phosphorylation in basic biological processes. Sawicka and colleagues investigated histone H3S28 phosphorylation (H3S28ph) in a mammalian system in the context of stress signaling. They found that H3S28ph is a hallmark of the transcriptional response to cellular stress [80]. In active host defense against pathogens and environmental insults, the inflammatory immune response requires coordinated activation of both transcription factors and chromatin to induce productive transcription. Allis and associates have recently identified H3S28ph as the principal stimulation-dependent histone modification in myeloid cells. They observed its enrichment at induced genes in mouse macrophages stimulated with bacterial lipopolysaccharide. They also identified mitogen- and stress-activated protein kinases (MSKs) as primary mediators of H3S28ph in macrophages [81]. Furthermore, rapid gene regulation in response to diverse environmental cues occurs in the context of chromatin condensation mediated by histone proteins. It has been shown that enriched integration of histone H3.3, the ancestral histone H3 variant, is a general feature of dynamic regulated chromatin and gene transcription. One key difference between this variant and ‘canonical’ H3.1 and H3.2 is that H3.3 contains a unique serine residue at position 31. Martire, Banaszynski and others have shown that phosphorylation of histone H3.3 at S31 promotes the activity of p300, an acetyltransferase, and enhancer of acetylation. This study demonstrates that a single amino acid in a histone variant can integrate signals and impact genome regulation globally [82]. In another study, Armache, Josefowicz and their team studied how phosphorylation plays a role in rapid gene induction. They showed that H3.3 is phosphorylated in a stimulation-dependent manner along rapidly induced genes in mouse macrophages. This selective mark of stimulation-responsive genes directly engages the histone methyltransferase SETD2, a component of the active transcriptional machinery, and excludes elongation corepressor ZMYND11. Thus, the authors propose this marked H3.3, provides preferential access to the transcription apparatus, one of the dedicated mechanisms for rapid gene induction [83].

Histone and related protein phosphorylation play important roles in cancer as well. It has been shown that histone H2A T120 phosphorylation promotes oncogenic transformation via upregulation of cyclin D1 [84]. The androgen receptor (AR) is critical for the progression of prostate cancer to a castration-resistant (CRPC) state. One study showed that the tyrosine kinase ACK1 phosphorylates histone H4 at tyrosine 88 upstream of the AR transcription start site. The WDR5/MLL2 complex reads the H4-Y88-phosphorylation marks and deposits the transcriptionally activating H3K4-trimethyl marks (H3K4me3) that promote AR gene transcription [85]. When H4-pY88 epigenetic marks were reversed using an ACK1 inhibitor, this sensitized naïve and enzalutamide-resistant prostate cancer cells expressing reduced AR levels, leading to slowed CRPC tumor growth.

Jumonji domain-containing 6 (JMJD6) is an epigenetic modifier that contains both arginine demethylase and lysine hydroxylase enzyme activities [86, 87]. In a recent study, the authors demonstrated that JMJD6 possesses intrinsic tyrosine kinase activity and can utilize ATP and GTP as phosphate donors to phosphorylate Y39 of histone H2A.X (H2A.X^Y39ph^). The JMJD6-H2A.X^Y39ph^) axis promotes growth of TNBC cells via an autophagy-dependent pathway. The authors also showed combined inhibition of JMJD6 kinase and autophagy efficiently inhibits TNBC growth [88].

Histone methyltransferase EZH2 is regulated by protein phosphorylation. Wan et al. have shown that AMP-activated protein kinase (AMPK) phosphorylates EZH2 at T311 to disrupt the interaction between EZH2 and SUZ12, another core component of the polycomb repressive complex 2 (PRC2), leading to attenuated PRC2-dependent methylation of H3-lys27 [89]. As the PRC2 target genes include a number of tumor suppressors, which may be upregulated upon EZH2-T311 phosphorylation, leading to suppressed tumor cell growth in vitro and in vivo.

### Small molecule inhibitors for epigenetic enzymes

Currently large numbers of small molecule inhibitors or activators of enzymes involved in epigenetic regulatory pathways are being developed for clinical translation. A number of epigenetic drugs have received regulatory agency approval for the treatment of human malignancies [40, 90] (Table 1). Examples of their chemical structures are presented in Table 2. The first two, the cytosine analogues 5-azacytosine (5-azaC; azacytidine) and 2′-deoxy-5-azacytidine (5-aza-dC; decitabine), have been studied in the treatment of myelodysplastic syndrome (MDS), a bone marrow disorder with a high risk for progression to AML [91]. 5-azaC (trade name Vidaza) was approved by the FDA for all five stages of MDS in 2004, which was quickly followed by approval of 5-aza-dC in 2006. These two drugs currently represent first-line therapies for MDS when stem cell therapy is not suitable. They have also been applied in the setting of chronic myelomonocytic leukemia (CMML) and AML.

**Table 1:** Chronical list of epigenetic drugs approved for cancer by the FDA and other authorities

Name	Year of approval	Mechanisms of action	Clinical applications
Azacitabine	2004	DNMT inhibitor	AML; CMML; MDS
Decitabine	2006	DNMT inhibitor	AML; CMML; MDS
Vorinostat	2006	HDAC inhibitor	Cutaneous manifestations of cutaneous T-cell lymphoma (CTCL)
Romidepsin	2009	HDAC inhibitor	CTCL and peripheral T-cell lymphoma (PTCL)
Belinostat	2014	HDAC inhibitor	Relapsed or refractory PTCL
Panobinostat	2015	HDAC inhibitor	Multiple myeloma
Chidamide^a^	2015	HDAC inhibitor	Relapsed/refractory PTCL
Enasidenib	2017	IDH2 inhibitor	Relapsed or refractory AML
Ivosidenib	2018	IDH1 inhibitor	Relapsed or refractory AML
Tazemetostat	2020	EZH2 inhibitor	Epithelioid sarcoma and relapsed or refractory follicular lymphoma

^a^China FDA’s approval

**Table 2:** Examples of small molecule epigenetic modifiers

Molecule name	Epigenetic target	Chemical structure
Azacytidine (5-Azacytidine)	DNMT1	
Vorinostat (SAHA)	Pan-HDAC	
Romidepsin (depsipeptide and SK228)	Class I HDACs	
Chidamide (HBI-8000)	Class I (HDAC1, 2, 3) Class IIb (HDAC10)	
GSK503	EZH2	
Tazemetostat	EZH2	
AS-8351	KDM5B (histone demethylase) inhibitor	
SIRT2104	SIRT1	
JQ1	BRD2, BRD3, BRD4, BRDT	
Onametostat [JNJ-64619178]	PRMT5 inhibitor	

Several HDAC inhibitors (HDACi) have also received FDA approval for clinical use. Vorinostat and romidepsin were the first drugs in this class initially approved for use in treating cutaneous T cell lymphoma (CTCL). SAHA, also known as Zolinza or Vorinostat, received FDA approval in 2006 and is currently a third-line therapy option for patients with CTCL [92]. Romidepsin was the second HDAC inhibitor approved by the FDA in 2009 [93]. Subsequently, Belinostat and Panobinostat, as well as Chidamine, were all approved for the treatment of relapsed or refractory peripheral T-cell lymphoma (PTCL) or multiple myeloma [94].

The third wave of FDA drug approval has occurred within the last few years. Enasidenib and Ivosidenib, inhibitors for isocitrate dehydrogenase 1 and 2 (IDH1/2), were approved for treating relapsed or refractory acute myeloid leukemia (AML) in 2017 and 2018, respectively [95]. Finally, in 2020, the FDA granted the accelerated approval to tazemetostat, a first-in-class inhibitor of the epigenetic writer “enhancer of zeste homolog 2” (EZH2), for the treatment of epithelioid sarcoma and relapsed/refractory follicular lymphoma [96].

## The epigenetics of the tumor microenvironment

The tumor microenvironment (TME) is composed of a broad range of cell types. Beyond tumor cells, the TME contains a variety of non-epithelial cell types, including those making up the blood vasculature (endothelial cells, pericytes and smooth muscle cells), cells involved with immune surveillance (lymphocytes, macrophages and mast cells), and stromal cells (i.e. fibroblasts), that are encompassed within the extracellular matrix (ECM) containing a range of diffusible growth factors, cytokines and chemokines [97–99]. It has long been recognized that carcinomas promote a modified stroma characterized by the expression of proangiogenic growth factors, altered ECM expression, accelerated fibroblast proliferation, and increased inflammatory cell infiltration. The dynamic interaction between cancer cells, non-cancer cells and non-cellular components determines whether carcinomas develop and progress in the immunocompetent host. As an example, stromal fibroblasts can have a profound influence on the development and progression of carcinomas [100].

Epigenetic alterations in the TME dictate tissue hypoxia and play a crucial role in the cellular response to hypoxia [101] and cancer cell metabolism [102]. Epigenetic regulators may work hand in hand with the hypoxia-induced transcription factor (HIF) family of genes in sustaining hypoxia-adapted cellular phenotypes long after their HIF-dependent initiation Epigenetic changes stabilize the binding of HIF their transcriptional targets, thereby impacting histone demethylase enzyme activity following direct HIF transactivation, and culminating in global patterns of histone modifications and DNA methylation in the hypoxic TME, with severe biological consequences [103–105]. Hypoxic upregulation of JMJD1A expression acts as a signal amplifier to facilitate hypoxic gene expression, ultimately enhancing tumor growth [106, 107]. Macrophages are key innate immune cells in the TME, where they regulate primary tumor growth, vascularization, metastatic spread and tumor response to interventional therapies. In macrophages, hypoxia-attenuated expression of Jumonji histone demethylase activity leads to increased histone H3K9 methylation and decreased chemokine expression, resulting in changes in the immune landscape within the TME [108, 109].

## Epigenetic regulation of the immune cells and immune-associated molecules

The immune system consists of a complex, integrated ensemble of organs, tissues, cells, and soluble mediators. Epigenetic mechanisms play key roles in immune cell differentiation and function, ensuring appropriate gene expression patterns in immune cells under different tissue microenvironmental conditions [10, 110, 111]. Importantly, epigenetic mechanisms play seminal roles in immune cells and stromal cell types within the TME.

Compelling evidence supports the notion that immune cells and their secreted mediators play dual roles in cancer development and progression [112, 113]. On one hand, normal immune surveillance or immunoediting are required for cancer prevention and the inhibition of tumor growth and progression. One the other hand, unresolved immune responses such as those occurring in cases of chronic inflammation can promote the growth and dissemination of cancer. In terms of mechanisms, the Th1 immune responses, including those mediated by cytotoxic CD8^+^ and CD4^+^ Th1 T cells, along with their characteristic Th1-associated cytokines, function as the major anti-tumor immune pathways restricting disease progression. In contrast, myeloid-derived suppressive cells (MDSC), pro-angiogenic Type-2 tumor associated macrophages (TAM) and/or their derivative cytokines IL-6, TNF, IL-1β and IL-23 are generally recognized as dominant tumor-promoters. Th17 cells and CD4^+^ CD25^+^ Foxp3^+^ regulatory T cells, and immunoregulatory cytokines such as TGF-β, may play equivocal roles in tumor development, depending on their context within the TME and triggering events leading to initial propagation of carcinogenesis [112].

**Fig. 2:**
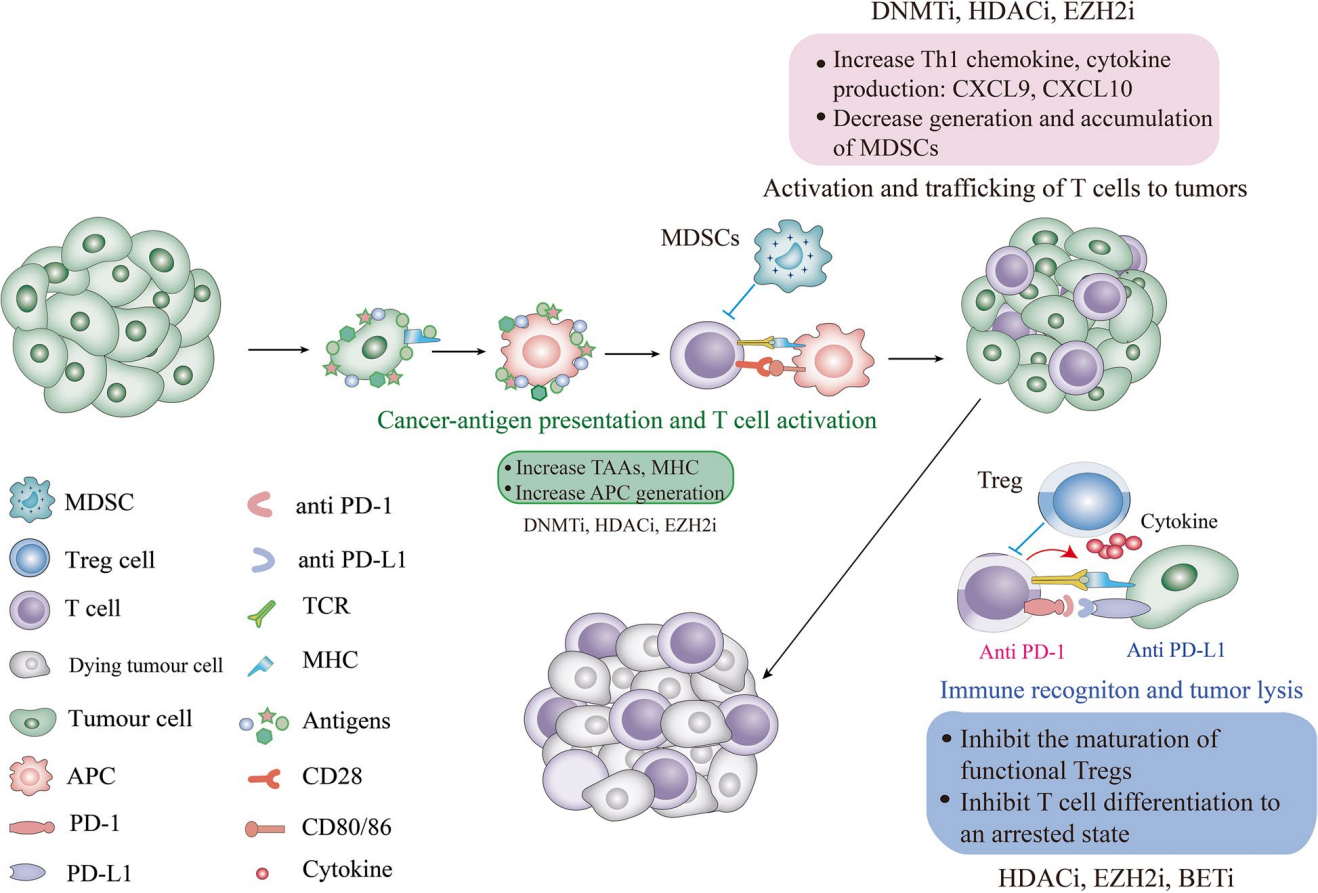
The potential functions of epigenetic modulators in multiple aspects of the TME and immune cycle. First, epigenetic drugs may induce ICD of cancer cells, enhance the expression of various tumor-associated antigens (TAAs), MHC molecules, and the generation of APC, thus enhancing immune cell priming and effector T cell recognition of tumor target cells. DNMTi, HDACi and HMTi (EZH2 and G9a) have demonstrated such biological effects [114–116]. Secondly, epigenetic drugs may target a variety of types of immune cells, resulting in reduced generation and accumulation of MDSC [117, 118], and inhibited differentiation and function of Treg (e.g., EZH2i) [119–121]. Third, during these processes, the drugs commonly result in compensatory increases in the production of effector T cells-chemokines and the activation of effector (anti-tumor) T cells, with therapeutic synergy observed for combined use with immune checkpoint blockade agents. The detailed effects of various classes of inhibitors have been discussed under various Sections. This figure is modified from Chen X. et al., 2020 [122]

In this section, we will discuss several key epigenetic mechanisms that impact key immune cell populations within the evolving TME and how these relate to disease outcomes (Fig. 2).

### Innate immune cells (myeloid cells and NK cells)

Myeloid cells play important roles in cancer cell recognition by the adaptive immune system and orchestrate the initiation of inflammation and protective anti-tumor immune responses [9, 123]. These cells include granulocytes, monocytes, macrophages, neutrophils, DCs, and MDSCs. In addition, we will include NK cells that are not of myeloid origin, but represent an important type of innate immune cell that both facilitates and mediates anti-tumor activity. We now discuss epigenetic mechanisms dictating the differentiation and function of each of these immune cell types.

#### Innate/myeloid immune regulation via DNA methylation

DCs initiate and orchestrate adaptive immune responses against infection and disease, and they are central to the development of immunologic memory and tolerance (to self). DCs rapidly integrate signals from their tissue microenvironments and respond accordingly to these signals, undergoing dramatic changes in transcriptional programming as a consequence of environmental stressors. This dynamic change relies on epigenetic changes in the chromatin structure of DCs as mediated by numerous enzymes and their substrates [124]. However, gene activation precedes DNA demethylation in response to infection in human DCs. This shows that DNA demethylation may play a limited role in establishing core regulatory DC programming after infection [125].

Cytokines play key roles in modulating specific transcriptional programs in innate immune cells. One standard method for ex vivo differentiation of DC from human monocytes is the use of a cocktail of cytokines including IL-4 and GM-CSF, whereas GM-CSF alone drives these cells to become macrophages. In a recent study, the authors found that IL-4 orchestrates TET-2-dependent, STAT6-mediated DNA demethylation leading to DC differentiation [126]. This is the first description of a cytokine-mediated event leading to direct gene-specific DNA demethylation in innate immune cell differentiation.

When human blood-derived monocytes differentiate into DCs, the cell surface expression of CD14 is lost whilst CD209 (aka DC-SIGN) expression is gained. These reciprocal changes are associated with the loss of epigenetic markers of “activation” at the CD14 locus, but the acquisition of the epigenetic markers at the CD209 locus. While there is little change in “repressive” histone marks and CpG methylation at the CD14 locus, these both occur at the CD209 locus. For repression of the active CD14 gene, the loss of “activation” histone modifications is likely necessary and sufficient for silencing. By contrast, the activation of the to that point silent CD209 gene appears to require the acquisition of “active” histone modifications and concomitant loss of both “repressive” histone marks and CpG methylation [127].

MDSCs are induced during neoplasia, where they mediate potent tumor-promoting activities [128]. DCs and MDSCs arise from common progenitors. TME-derived factors redirect progenitor differentiation away from immune-promoting DCs and towards tolerogenic/suppressor MDSCs, representative of one of the immunological hallmarks of cancer. Studies have demonstrated in vitro differentiation of DCs from human primary monocytes may result in the generation of MDSCs in the presence of PGE_2_ or tumor-associated media. Comparison of the DC vs. MDSC DNA methylome has revealed extensive demethylation in the genome, with specific gains of DNA methylation and repression of immunologic gene signatures in MDSCs. This process was association with DNMT3A levels. Hence, this study links PEG_2_- and DNMT3A-dependent DNA hypermethylation with the development of MDSC and their immunosuppressive functions [129].

NK cells play important roles in immune surveillance and the elimination of stressed, infected or transformed cells. In addition to their classic well-defined functions as spontaneously-active cytotoxic effector cells, a recent study found that NK cells also serve as recruiters of cDC1 into the TME in association with increased immune-mediated control of cancer growth [130]. While chronic antigen stimulation drives the proliferation of CD8^+^ memory T cells in association with genome-wide epigenetic reprograming and dysfunction, the authors showed that chronic stimulation of NK cells through NKG2C using plate-bound agonistic antibodies in combination with IL-15 drove robust proliferation and activation of CD3^neg^CD56^dim^CD57^+^NKG2C^+^ NK cells while simultaneously inducing high levels of expression of the checkpoint inhibitory receptors LAG-3 and PD-1. Chronically-stimulated NK cells were rendered dysfunctional when re-challenged with tumor targets, with these anergic NK cells displaying a specific pattern of epigenetic reprograming with genome-wide alterations in DNA methylation [131]. However, depending on the status of PD-1 expressed by such exhausted NK cells, these effector cells may be rescued via the application of immune checkpoint blockade (i.e., anti-PD-1, anti-PD-L1).

#### Innate/myeloid immune regulation via histone modifications

The activation and maturation of DC is modulated by histone modifications. For example, FOXM1 represses the maturation of bone-marrow-derived DCs (BMDCs), in association with decreased IL-12 production, and the inability of DCs to promote T-cell proliferation in tumor-bearing mice. Notably, the expression of FOXM1 is epigenetically regulated by demethylation on H3 lysine 79 (H3K79me2). Furthermore, inhibition of the H3K79 methyltransferase DOT1L leads not only to decreased enrichment of H3K79me2, but also to reduced FOXM1 expression, partially reversing its immunosuppressive effects on BMDCs [132].

Monocytes attracted by tumor-induced chronic inflammation may differentiate into APCs, the type of which depends on cues in the TME. Epigenetic mechanisms are involved in the regulation and functional polarization of TAMs within tumor tissues [109]. Van der Burg and colleagues showed that human cervical cancer cells either hampered the differentiation of monocytes into DC, or they skewed differentiation towards pro-angiogenic/pro-tumorigenic M2-like macrophages [133]. Interestingly, depletion of TAMs switches the epigenetic profile of tumor-infiltrating T cells (TILs) towards Type-1 functional polarity and restores their anti-tumor phenotype in murine pancreatic carcinoma models [134].

In macrophages, their functional polarization state requires precise temporal and situational regulation of target-gene expression. Epigenetic changes play roles in altered cell signaling and signature gene profiles during M1 and M2 polarization [135, 136]. Nguyen et al. studied how to eliminate antigen-loss variant tumor cells after adoptive T cell therapy applied in combination with oncolytic virus vaccination [137]. The authors showed that tumor relapse from the aforementioned therapy can be prevented using class I HDACi, MS-275. Drug administration subverted the phenotype of tumor-infiltrating CD11b^+^ Ly6C^hi^ Ly6G^−^ myeloid cells, favoring NOS2/ROS secretion and expression of pro-inflammatory genes characteristic of M1 polarization. Mechanistically, MS-275 abrogated the immunosuppressive function of tumor-infiltrating myeloid cells and reprogrammed these cells to eliminate target antigen-deficient tumor cells in a caspase-dependent manner. The data suggests that MS-275 modulates the local cytokine landscape to favor anti-tumor myeloid cell polarization via an IFN-γR/STAT1 signaling axis.

Many studies have shown that epigenetic pathways of histone modifications regulate various aspects of MDSCs. For example, HDAC11 is a novel epigenetic regulator of cell expansion and function in tumor-associated MDSCs [138]. In another study, the authors showed that epigenetic component p66a modulates MDSCs by modifying the activity of STAT3 [139]. Interestingly, p66a expression was significantly suppressed by IL-6 both in vitro and in vivo during MDSC activation, suggesting that p66a is involved in IL-6–mediated differentiation of MDSCs. Finally, inhibition of EZH2 by GSK126 has been shown to suppress antitumor immunity by reinforcing MDSC content in tumors [140].

HDACi exerts a range of effects on MDSCs. One early study showed that TSA facilitates GM-CSF-mediated expansion of MDSCs in vitro and in vivo [141]. However, later studies showed that several HDACi delete or inhibit MDSCs in tumors. Wang et al. showed that HDACi SAHA eliminates MDSCs in the 4 T1 breast carcinoma model by inducing apoptosis of Gr1^+^ cells [142]. HDACi CG-745 can also reduce MDSCs content, thereby promoting anti-tumor immunity within the TME of CT26 colon cancer in mice [143]. In another study involving epigenetic therapy, treatment with HDACi and DNMTi resulted in significant reductions in tumor-associated MDSCs [117]. HDACi trichostatin-A has also been reported to inhibit the recruitment of MDSC into the TME and to potentiate the anti-tumor activity of macrophages in several translational tumor models [118].

For NK cells, the H3K4me3 demethylase Kdm5a is required for cell activation to suppress SOCS1 through association with p50 [144]. Another recent study claimed that a discrete subset of epigenetically-primed human NK cells mediates the development of antigen-specific immune responses [145], although the specific underlying epigenetic mechanism of action remains unknown.

### CD4^+^ T cells

The differentiation of CD4^+^ T helper cells is controlled by ‘master’ transcription factors that commit naïve T cells to become a Th1 (Tbx21), Th2 (GATA3), Th9 (PU.1), Th17 (RORC), Follicular helper T (Tfh cells) cells (BCL6), or a Treg (Foxp3), each of which has a distinct cytokine secretion profile that reinforces or restricts innate and adaptive effector cell functions. Cytokine production by various innate cells influences CD4^+^ T cell effector cell programming. The bifurcated, parallel axes allow CD4^+^ T cells flexibility to adjust their functional polarity to prevent (or exacerbate) disease [146]. Th1 cells produce IFN-γ and provide protection against intracellular pathogens and cancer. Th2 cells produce cytokines IL-4, IL-5, and IL-13, which stimulate B-cell antibody production and are they are also involved in host defense against parasites [147]. Th17 cells produce the cytokine IL-17 as well as IL-21 and IL-22, and are involved in neutrophil-mediated protection against extracellular bacteria, where they link innate and adaptive immunity [148].

It has been well documented that epigenetic mechanisms play key roles in the cell differentiation and functions of Th1 and Th2 cells [149]. STAT4 and STAT6 transcription factors play discrete roles in tuning epigenetic modifications and transcription during T helper cell differentiation [150]. Using chromatin immunoprecipitation and massive parallel sequencing, Wei et al. quantitated the full complement of STAT-bound genes, and concurrently assessed global STAT-dependent epigenetic modifications and gene transcription by using cells from cognate STAT-deficient mice. They found that, globally, STAT4 had a more dominant role in promoting active epigenetic marks, whereas STAT6 had a more prominent role in antagonizing repressive marks [150]. As for each subset of CD4^+^ T cells, epigenetic mechanisms are involved in the regulation of key genes and key cytokine production or silencing. EZH2 and histone 3 trimethyl lysine 27 have been associated with Il4 and Il13 gene silencing in Th1 cells [151]. For Th2 cells, epigenetic regulations have been involved in the induction, maintenance, heterogeneity, and recall-response of effector and memory functions [152]. Epigenetic changes at the Gata3 gene locus are essential for the acquisition and maintenance of the Th2 cell identity [152, 153].

Transforming growth factor-β (TGF-β) functions as a regulatory ‘switch’ that in combination with other cytokines can ‘reprogram’ effector T cell differentiation along different pathways. It reprograms the differentiation of Th2 cells and promotes an IL9–producing subset, Th9 cells [154]. In this case, EGFR-HIF1α signaling positively regulates the differentiation of IL-9 producing Th9 cells [155], and HDAC SIRT1 negatively regulates the differentiation of IL-9-producing CD4^+^ T cells [156]. Th9 cells can be generated by treatment of naive T cells with TGF-β and IL-4 in vitro. Smad2 and Smad4, two transcriptional factors activated by TGF-β signaling, are required for Th9 differentiation in vitro. Deficiency of Smad2 or Smad4 in T cells resulted in impaired IL-9 expression, which was coincident with enrichment of repressive chromatin modification histone H3 K27 trimethylation and enhanced EZH2 binding to the Il9 locus. Thus, the TGF-β:Smad2/4–signaling pathway regulates IL-9 production through an epigenetic mechanism [157]. Another study identified age- and differentiation status–related epigenetic modifications of PU.1 promoter region as a unique regulator of Th9 memory acquisition and Th9 immunity [158]. As for Th17 cells, not too surprisingly, epigenetic modifications as a mechanism for regulating IL-17 production is suggested in Th17 cell differentiation [159]. In addition, the fate of Th17 Cells is dictated by epigenetic modifications and also remodeled by the TME [160]. A recent study demonstrated that IL-17-producing cells promote terminal exhaustion of CD8^+^ T cells and tumor progression in vivo, which can be reversed by blockade of IL-17 or suppression of the RORγt pathway [161]. This provides another target for epigenetic modulation in cancer immunotherapy.

For Tfh cells, it has been demonstrated that the VHL-HIF-1α axis played an important role during the initiation of Tfh cell development through glycolytic-epigenetic reprogramming [162]. Ezh2 is an HMT that catalyzes H3K27me3 and impacts Th1, Th2 and Treg cells primarily via HMT activity. The authors also showed that Ezh2 ablation impairs T follicular helper (Tfh) cell differentiation and the activation of Tfh transcriptional programming [163]. Mechanistically, Ezh2 is recruited by Tcf1 to directly activate Bcl6 gene transcription, and this function requires Ezh2 phosphorylation at Ser21. Meanwhile, Ezh2 deploys H3K27me3 to the Cdkn2 gene promoter region where it represses its expression in Tfh cells, leading to aberrantly upregulated p19Arf triggering Tfh cell apoptosis and antagonized the function of Bcl6.

### Treg cells

The development of Treg cells is critically dependent on X-linked transcription factor forkhead box P3 (FoxP3). These cells are characterized by sustained expression of FOXP3 and they play crucial roles in maintaining immune system homeostasis. Foxp3 programs both the development and function of CD4^+^CD25^+^ regulatory T cells (Treg cells) [164, 165]. The expression of FoxP3 is required for optimal Treg suppressor activity, with Treg cells dependent on the presence of paracrine IL-2 [166]. Work over the past several decades revealed that the DNA methylation of the CpG island in the enhancer region controls expression of *FoxP3* in T cells [167–169]. Human γδ T cells are potent cytotoxic effector cells, producing a variety of cytokines, which can also acquire regulatory activity. Interestingly, vitamin C promotes conversion of human γδ T cells into FOXP3^+^ Treg cells via a mechanism involving epigenetic regulation [170]. Specifically, phospho-modified Vitamin C induces the hypomethylation of the FOXP3 gene promoter region in Treg cells.

TET methylcytosine dioxygenases also appear essential for the functional stability of Treg cells. Yue, Rao and colleagues conducted a series of studies demonstrating key roles for TETs in maintaining stable FoxP3 expression in Treg cells. First, they showed that during Treg development in the thymus, TET proteins mediate the loss of 5mC in Treg cell-specific hypomethylated regions and intronic cis-regulatory elements in the Foxp3 locus. The stability of Foxp3 expression is markedly compromised in Treg cells from Tet2/Tet3 double-deficient mice. Vitamin C potentiates TET activity and acts through Tet2/Tet3 to increase the stability of Foxp3 expression in TGF-β-induced Treg cells [171]. In a second study, Tet2/3 fl/flFoxp3Cre mice lacking Tet2 and Tet3 in Treg cells were shown to develop inflammatory disease, with Treg cells from these mice exhibiting altered Treg gene signatures, with an associated upregulation in the transcription of genes involved in cell cycle, DNA damage and cancer. In littermate mice with severe inflammation, both CD4^+^Foxp3^+^ and CD4^+^Foxp3^−^ cells show strong skewing towards Tfh/Th17 phenotypes. These results indicated that Tet2 and Tet3 are guardians of Treg cell stability and immune homeostasis [172]. Finally, the authors performed whole-genome analyses and showed that the transcriptional program and epigenetic features of Treg cells are attenuated in the absence of Tet2 and Tet3. The addition of the TET activator vitamin C during TGFβ-induced iTreg cell differentiation in vitro potentiates the expression of Treg signature genes and alters the epigenetic landscape to better resemble Treg cells generated in vivo [173].

The importance of EZH2 in Treg cells has also been demonstrated. Yang et al. have shown that EZH2 is crucial to both the differentiation of Treg and the expansion of T effector cells [174]. DuPage et al. showed that Ezh2 is critical for the maintenance of Treg cell identity after activation [175]. Disruption of EZH2 activity in Treg cells via either genetic or pharmacologic means led to the acquisition of pro-inflammatory functions in tumor-infiltrating Treg cells, the remodeling the TME and enhanced recruitment and function of CD8^+^ and CD4^+^ effector T cells, leading to tumor elimination [119]. In another study, genetic depletion of EZH2 in Treg cells led to robust anti-tumor immunity in mouse models [120].

In one study, a second generation BETi, PLX51107, has been shown to reduce the tumor-infiltrating Treg in a murine melanoma model [176].

### CD8^+^ T cells

Epigenetic regulation of gene expression plays a key role in the acquisition and maintenance of effector function in CD8^+^ T cells, and in the rapid and robust response of memory CD8^+^ T cells to re-challenge with antigen [10, 177, 178]. Epigenetic mechanisms also impact stemness in CD8^+^ T cells and their fate [179]. Notably, these foundational observations have been confirmed and extended greatly using cutting-edge technologies, such as single-cell RNA sequencing [8, 180].

Memory CD8^+^ T cells are capable of rapidly producing high levels of pro-inflammatory cytokines, killing target cells, and proliferating and differentiating into secondary effectors within days of re-exposure to cognate antigen. This response-ready state contributes to the superior ability of this cell type to confer protective immunity against infectious agents and cancer. However, the underlying mechanisms responsible for such operational readiness remain incompletely resolved, although epigenetic mechanisms are clearly involved. CD4^+^ T helper-dependent chromatin remodeling provides a molecular basis for the enhanced responsiveness of memory CD8^+^ T cells [181]. Histone acetylation facilitates rapid and robust memory CD8^+^ T cell response through differential expression of effector molecules: eomesodermin and its targets, perforin and granzyme B (GzmB). Accessible chromatin associated H3 lysine 9 acetylation (H3K9Ac) was found to be significantly higher at the proximal promoter and the first exon region of all three genes in memory CD8^+^ T cells vs. naive CD8^+^ T cells [178]. Genome-wide analysis of histone methylation pattern reveals chromatin state-based regulation of gene transcription and function of memory CD8^+^ T cells [182]. Thus, epigenetic changes mediated via histone acetylation and methylation may provide chromatin “memory” for the rapid and robust transcriptional responses associated with memory CD8^+^ T cells.

HDAC3 has been identified as an epigenetic regulator of CD8^+^ T cell effector differentiation and cytotoxic potential. HDAC3 inhibits CD8^+^ T cell cytotoxicity early after activation but is required for the persistence of activated CD8^+^ T cells following resolution of acute infections [53]. Mechanistically, HDAC3 inhibits genetic programs associated with differentiation of CD8^+^ T cells and their cytotoxic activity.

### Bioactive immune molecules

At the molecular level, key effector molecules associated with immune functions (STING), function of CD8^+^ cytotoxic T cell (GzmB, interferon-γ, IL-2, IL-12) and FOXP3^+^ Treg cells are known to be regulated via epigenetic pathways (Table 3). Indeed, many cytokines and chemokines are regulated via epigenetic pathways in cancer [183].

**Table 3:** Epigenetic regulation of key molecules in immune and cancer cells

Molecule	Cell types	Epigenetic regulation	Reference
Granzyme B	CD8+ T cells	H3K9Ac at the promoter increased ~ 100-fold within 12 h stimulation	[184]
IFN-γ	CD8+ T cells (naïve and memory)	Promoter DNA methylation downregulates transcription in naïve cells, while demethylation happens rapidly in memory cells leading to IFN-γ expression	[177]
IL-2	CD8+ T cells	1. The promoter-enhancer region is demethylated following T cell activation. 2.Activated Ag-specific CD8^+^ T cells exhibit rapid DNA demethylation at the I-2 locus and is maintained through memory development	[185–187]
IL-12	T, NK and macrophages	H3K4me3 up-, H3K27me2 down-regulate IL12p35 and IL12p40 promoters.	[188]
FoxP3	Treg	DNA methylation of the CpG island in the enhancer region dictates its expression in T cell subsets.	[167, 169]
STING	Cancer cells	Promoter hypermethylation of *cGAS* and *STING* genes mediated their coordinate transcriptional silencing, and DNA methylation inhibitor can restore their expression.	[189]
PD-1, CTLA-4, TIM-3, LAG-3, TIGIT and PD-L1	Cancer and stromal cells	DNA methylation and H4K9 and H3K27 trimethylation are associated with silenced status	[190–192]

#### STING

STING is a master regulator of cancer immunity [193, 194]. STING functions in the TME can enhance the development of therapeutic tertiary lymphoid structure [195]. STING signaling may be commonly suppressed in a greater variety of tumors due to loss-of-function mutation or epigenetic silencing of the STING/cGAS promoter regions [196]. In fact, histone demethylases KDM5 repress immune response via suppression of STING [197]. In human tumors such as human papilloma virus (HPV)-positive head and neck cancer, KDM5B expression is inversely correlated with STING expression, with the level of intratumoral CD8^+^ T cells, and with patient survival in cancers with a high level of cytosolic DNA [197]. STK11 (Liver kinase 1, LKB1) was first identified as a tumor suppressor gene through its association with Peutz-Jeghers Syndrome. Recently, it was discovered that suppression of STING was associated with LKB1 loss in KRAS-driven lung cancers. This effect was mediated in part by hypermethylation of DNMT1 and EZH2 activity related to elevated S-adenylmethionine levels reinforced by DNMT1 upregulation [198]. In human melanoma, promoter hypermethylation of cGAS and STING genes mediates their coordinated transcriptional silencing and contributes to the widespread impairment of the STING signaling function [189]. The authors demonstrated that this suppression is reversible through pharmacologic inhibition of DNA methylation. Demethylation-mediated restoration of STING signaling could improve their antigenicity through the up-regulation of MHC class I molecules and thereby enhance their recognition and killing by CTLs [189].

#### GzmB

GzmB is a serine protease that serves as an important mediator of target-cell apoptosis mediated by immune cells, such as NK cells and cytotoxic CD8^+^ T cells. Juelich et al. examined the epigenetic control of GzmB expression in murine polyclonally-activated CD8^+^ T cells as they differentiate from naïve to effector phenotypes following in vitro stimulation with mitogenic anti-CD3/CD28 Abs [184]. Following in vitro activation, both CD4^+^ and CD8^+^ T cells exhibit rapid histone H3 loss at the granzyme B (gzmB) gene proximal promoter region. However, despite this promoter being remodeled in both T cell subsets, only CD8^+^ T cells express high levels of gzmB and display a distinct pattern of key epigenetic marks, notably differential H3 acetylation and methylation. One key epigenetic marker occurs within 12 h of stimulation, i.e. H3K9Ac modification at the *gzmB* promoter is increased ∼100-fold in CD8^+^ T cells but remains unchanged in the CD4^+^ T cells.

#### IFN-γ

IFN-γ is a key cytokine associated with Type-1, pro-inflammatory immune responses against cancer cells. The CpG methylation in the promoter region regulate IFN-γ gene expression in naïve and in vitro-activated murine CD8^+^ T cells [199]. In clonal populations of primary cells, the *IFNG* proximal promoter in naïve cells is heavily methylated, with cell activation leading to rapid demethylation of the promoter region in some, but not all clones. Kersh and others have extended these observations investigating adoptive transfer of LCMV-specific TCR transgenic CD8^+^ T cells and LCMV infection to show that memory populations reacquire a methylated promoter profile, characteristic of naïve cells, which correlates with reduced production of IFN-γ [200]. Importantly, demethylation of the *IFNG* locus occurred far more rapidly in memory vs. naive T cells. Thus, DNA methylation can negatively regulate IFN-γ expression in CD8^+^ T cells [177].

The expression of IFN-γ by CD4^+^ T cells is observed only after Th1 cell differentiation. However, while naive CD8^+^ T lymphocytes fail to produce large amounts of IFN-γ, after TCR stimulation, there is a progressive acquisition of IFN-γ production as these cells differentiation into cytotoxic T lymphocytes (CTL) and memory cells. Finally, epigenetic therapies may activate type I interferon signaling in murine ovarian cancers leading to reduced immunosuppression and a reduction in tumor burden [117].

#### IL-2

The demethylation of CpG sites in the IL-2 gene promoter region proceeds by an active process and leads to enhanced transcription of the gene, and demethylation of a specific CpG site, providing an additional level of functional epigenetic memory [185, 186]. Thomas et al. showed that co-stimulation of T cells through CD28 led to marked, stable histone acetylation and loss of cytosine methylation at the IL-2 promoter/enhancer regions. This was accompanied by extensive remodeling of the chromatin in this region to a structure highly accessible to DNA binding proteins [201]. It is interesting to note that the epigenetic remodeling of the IL-2 locus in memory CD8^+^ T cells is influenced by CD4^+^ T cells. Activated Ag-specific CD8^+^ T cells exhibit rapid DNA demethylation at the IL-2 locus which is maintained throughout development towards memory cells [181, 187]. Histone dynamics on the promoter region also takes place during T cell activation or suppression. In response to T cell activation, there was an apparent decrease of histone acetylation and phosphorylation signals at the proximal promoter region of the inducible IL-2 gene [202]. This apparent decrease was due to a loss of binding histone H3 and H4 proteins, corresponding to a decrease in nucleosome occupancy at the promoter. This histone loss was found to be reversible and was dependent on the continual presence of appropriate activating signals and transcription factors, but not the acetylation status of the histone proteins [202]. One way for TGF-β to regulate immune activity is via the suppression of IL-2 production from T cells. In a recent study [203], the authors demonstrate that Smad2 and Smad3, two major TGF-β-downstream transcription factors, are redundant, but essential for TGF-β-mediated suppression of IL-2 production in CD4^+^ T cells. Both Smad2 and Smad3 were recruited into the proximal region of the IL-2 promoter in response to TGF-β, with H3K9 trimethylation found to be increased in the proximal region of the IL-2 promoter, which occurred in a Smad2/3-dependent manner. The H3K9 methyltransferases Setdb1 and Suv39h1 bound to Smad3 and suppressed IL-2 promoter activity in collaboration with Smad3. The authors proposed that Smads recruit H3K9 methyltransferases Suv39h1 to the IL-2 promoter, thereby inducing suppressive histone methylation and the inhibition of T cell receptor-mediated IL-2 transcription.

IL-2 itself modulates the regulatory T cell epigenetic landscape [204]. IL-2 regulates the positioning of the pioneer factor SATB1 in CD4^+^ thymocytes and controls genome-wide chromatin accessibility in thymic-derived Treg cells. These findings may have broad implications for potential therapeutic strategies to reprogram Treg cells in vivo.

#### IL-12

IL-12 is a heterodimeric cytokine that acts as a growth factor for activated T and NK cells, enhances the lytic activity of NK/lymphokine-activated killer cells, and stimulates the production of interferon-*γ* by resting PMBC. One of the key hallmarks for alpha-type-1 polarized DCs with optimized CTL-inducing activity is their high level of IL-12 production [205]. The level of IL-12 production also serves to discriminate macrophage functional polarity states. Hence, high levels of IL-12 and low levels of IL-10 production are features of M1 macrophages, while low levels of IL-12 and high levels of IL-10 production are features of M2 macrophages [206].

In a sepsis model, investigators have shown that deficiency in IL-12 production by DC was due to epigenetic alterations [188]. The suppression of DC-derived IL-12 persisted for at least 6 weeks and was not due to the action of immunoregulatory cytokines. Specifically, IL-12p70 expression was regulated by stable reciprocal changes in histone H3 lysine-4 trimethylation (H3K4me3) and H3 lysine-27 dimethylation (H3K27me2), as well as changes in cognate histone methyltransferase (HMT) complexes on the *Il12p35* and *Il12p40* promoters. This study implicates histone modification enzymes in suppressing IL-12 gene product expression in DC, which may represent principal mechanisms underlying long-term immunosuppression subsequent to response to sepsis.

#### Checkpoint molecules in immune and stromal cells

Immune checkpoint molecules (ICMs) include programmed cell death protein 1 (PD-1), cytotoxic T-lymphocyte-associated protein 4 (CTLA-4), T cell immunoglobulin and mucin-domain containing-3 (TIM-3), lymphocyte-activation gene 3 (LAG-3) and T cell immunoreceptor with Ig and ITIM domains (TIGIT). The expression of these molecules and their receptors on the surface of cancer cells and immune cells, or their soluble, secreted forms can create an immune-subversive TME that helps tumor cells to evade immune destruction [190, 207]. Epigenetic mechanisms play critical roles in regulating the expression of ICMs and their receptors in the TME [190, 208]. As a specific example, LAG-3 DNA methylation correlates with expression of this immune checkpoint molecule in tumor and immune cells, impacting the fate of immune cell infiltrates in clear cell renal cell carcinoma [209].

Indeed, expression of PD-1, CTLA-4, PD-L1 and PD-L2 are enhanced by DNA hypomethylating agents in CD34^+^ cells from patients with myelodysplastic syndrome [210]. HDAC inhibition upregulates both PD-L1 and PD-L2 in melanoma [211]. A recent study found DNA methylation and repressive H3K9 and H3K27 trimethylation in the promoter regions of the genes for PD-1, CTLA-4, TIM-3, LAG-3, TIGIT, and PD-L1 in human breast cancer [191] and colorectal cancer [192]. Earlier, another group has analyzed epigenetic modifications of PDCD1 (PD1), CD274 (PD-L1), and CTLA4 in NSCLC tissues, and found that decreased methylation of regulatory regions in CTLA 4 and PDCD1 (PD1) genes correlated with increased expression of these ICM in the TME of NSCLC [212], suggesting the utility of these epigenetic modifications as potential diagnostic/prognostic biomarkers and/or therapeutic targets in the cancer setting.

As epigenetic mechanisms are involved in modulating immune functions of stromal and immune cells in the TME [213], it is highly likely that ICM expressed by stromal cells is also regulated via epigenetic mechanisms. Indeed, Fu and colleagues have shown that PD-L1 expressed on host cells is essential for PD-L1 blockade–mediated tumor regression [214]. In this light, epigenetic strategies have been developed and explored for their ability to synergize with PD-L1/PD-1 targeted cancer immunotherapies for enhances antitumor responses [122].

## Epigenetic and metabolic reprogramming of cancer and immune cells

Metabolic reprogramming of cancer cells represents a well-established hallmark of cancer [97, 215, 216], and this has emerged as a key immunosuppressive mechanism in modulating anti-tumor immune responses. In both cases, metabolic reprogramming and epigenetic reprogramming are interconnected, and to a large extent, metabolic state dictates epigenetics in cancer [217].

We have begun to understand the multiple roles that metabolic state dictates innate immune cell function and fate [218]. Metabolic pathways such as glycolysis or oxidative phosphorylation regulate macrophage function during inflammation and tissue repair. Activation of macrophages and DCs by pro-inflammatory stimuli causes them to undergo a switch toward glycolysis and away from oxidative phosphorylation, similar to the Warburg effect associated with cancer cells. Alpha-ketoglutarate orchestrates macrophage activation to M2 phenotype through metabolic and epigenetic reprogramming [219]. In another study, the authors demonstrated that VHL deficiency reinforces a state of glycolytic metabolism, leading to decreased respiratory capacity and reduced osteopontin expression in alveolar macrophages, resulting in the impaired function of type 2 innate lymphoid cells via a signaling cascade mitigated by HIF1α inhibition or its genetic ablation. Enhanced glycolysis was also determined to impact the epigenetic modification of osteopontin gene expression, with the metabolic intermediate 3-phosphoglyceric acid serving as a key checkpoint controller [220].

Additional studies have led to a better understanding of how metabolism regulates T cell differentiation, function and fate. It has been known that naïve T cells depend primarily on the oxidation of fatty acids as a primary source of energy. Upon cognate antigen recognition, T cells shift to glycolysis to sustain their effector function. Restifo, Gattinoni and colleagues found that by inhibiting glycolytic metabolism they could enhance immunologic memory as well as the anti-tumor function(s) of CD8^+^ T cells [221]. Hermans et al. have shown that lactate dehydrogenase inhibition synergizes with IL-21 to promote CD8^+^ T cell stemness and antitumor immunity [222]. Accumulating evidence further suggests that metabolism impacts cellular signaling and epigenetics to govern the longevity of T cells [223]. Finally, both metabolism and epigenetics regulate T cell exhaustion [224]. Exhausted T cells undergo metabolic insufficiency with altered signaling cascades and epigenetic landscapes that dampen effector immunity, leading to poor host responsiveness to immunotherapy. How metabolic stress affects T cell exhaustion remains an active area of investigation [224]. Finally, conditions within the TME reinforce epigenetic reprogramming of both cancer cells and immune cells, leading to an immunosuppressive millieu, however, these epigenetic modifications exhibit plasticity that may be corrected with epigenetic modulators [225].

## Modulation of anti-tumor immunity using epigenetic-targeted drugs

Epigenetic drugs have often been used in combinations with other immunostimulatory agents to achieve five goals: 1.) intrinsic cancer cell growth arrest, 2.) induction of tumor cell death (via apoptosis, necrosis, autophagic cell death), 3.) promoting expression of tumor-associated neoantigens for improved immune cell recognition [19, 226], 4.) reversal of hypoxia and inhibition of tumor angiogenesis, and 5.) modulation of immune cell (DC, T cells, among others) function in the TME.

We will focus on recent studies related to DC and T cells, two TYPES OF immune cells critical to the adaptive immune response to cancer.

### Tumor immunogenic cell death (ICD) and the activation of innate immune cells

ICD is a type of cell death that results in tumor cell destruction and the release of potent danger signals and tumor-associated antigens capable of promoting antitumor immune responses in vivo [227]. These danger signals, DAMPs, consist of “find-me signals” such as extracellular ATP and HMGB-1, and “eat-me signals”, such as ecto-calreticulin on the cell surface [228]. These danger signals activate DCs, which acquire elaborated tumor antigens and become activated to a more mature/stimulatory state that is optimal for the initiation of antitumor immune responses. Studies have found that many epigenetic drugs can function as ICD inducers when administered to cancer cells [114], such as valproic acid and vorinostat [229].

One study examined the effects of 5-aza on human DCs in vitro, and the resultant type of immune response induced in patients after treatment with 5-aza [107]. CD40 and CD86 costimulatory molecule expression were significantly increased on mature DC exposed to 5-aza (5-aza-DC). While mature DC production of IL-6, IL-12p70, IL-23 and TNF-α were unaffected by treatment with 5-aza, DC conditioned by 5-aza secreted significantly lower levels of IL-10 and IL-27 vs. control mature DC. In patients with advanced-stage myeloid malignancies, treatment with 5-aza led to a significant decrease of IL-4 secreting CD4^+^ T cells, and a significant increase of IL-17A- and IL-21-secreting CD4^+^ T cells in the peripheral blood. These results suggested that a Th17 response pattern was induced in patients receiving 5-aza treatment. In all, these data suggest potentially novel mechanisms of action of epigenetic agent-based therapies, which may have broader implications for the development of superior combination immunotherapeutic strategies.

Another clinical study reported that treatment with low-dose combinations of two FDA-approved epidrugs, azacytidine (A) and romidepsin (R), along with IFNα2 (ARI) hampers the aggressiveness of colorectal carcinoma cells and cancer stem cells in vivo and triggers tumor cell ICD that in turn, stimulates DC function [230]. The authors found that this triple drug combination increased accessibility of regulatory sequences in ISGs and IRFs promoters that had previously been epigenetically silenced in both colorectal cancer cells and DCs. Likewise, specific ARI-induced histone methylation and acetylation changes marked epigenetically affected ISG promoters in both metastatic cancer cells and DCs. ChIP-seq analysis confirmed such ARI-induced epigenetic changes impacted the IFN signature. Furthermore, the activation of this signal endowed DCs with a marked migratory capability, required for T cell crosspriming in tumor draining lymph nodes [231].

### Inhibition of immunosuppressive cells (Treg, MDSCs and TAMs)

HDACi can exert positive or negative effects on Treg cells, thus careful consideration is required prior to the administration of a particular HDACi in order to achieve a preferred immunogic outcome. Tao et al. found that HDACi treatment increased Treg expression of CTLA-4, GITR and PD-1, while maintaining repression of IL-2 in T*reg* cells. This resulted in increased numbers of T*reg* cells in vivo, with more potent suppressive activity against conventional (non-Treg) T effector cells [232]. Although Treg express multiple HDACs, HDAC9 has proven particularly important in regulating Foxp3-dependent suppression. Optimal Treg function requires acetylation of several lysines in the forkhead domain of FOXP3, and FOXP3 acetylation enhanced binding to the IL-2 gene promoter and consequent suppression of endogenous IL-2 production. HDACi can impair innate immune cell responses to Toll-like receptor agonists and to infection [233]. Furthermore, this therapeutic approach might be useful in lowering the incidence or severity of autoimmune diseases and transplant rejection via its immunoregulatpry action [232, 234].

Conversely, other HDACi have been reported to be immunostimulatory. A class I specific HDACi, entinostat, when applied at low doses, down-regulates Foxp3 transcription/expression in Treg, resulting in loss of suppressive function in Treg, without affecting intrinsic T effector cell activity [235]. When applied in vitro, low doses of entinostat induced STAT3 acetylation and corollary repression of FOXP3 in Treg cells [235].

EZH2 mediates epigenetic regulation of T cell differentiation and Treg function. Three recent studies have investigated the potential of using inhibitors of EZH2 to enhance cancer immunity and immunotherapeutic efficacy. In one study, Wang et al. showed that disruption of EZH2 activity in Tregs, using the EZH2 inhibitor CPI-1205, led to Treg cell acquisition of pro-inflammatory functions in the TME, with a coordinate increase in the recruitment and function of therapeutic CD8^+^ and CD4^+^ effector T cells [119]. In another study, the authors also showed that pharmacological inhibition of EZH2 in human T cells using CPI-1205 elicited phenotypic and functional alterations of the Tregs and enhanced cytotoxic activity of effector T cells. Moreover, anti-CTLA-4 abs increased EZH2 expression in peripheral T cells isolated from treated patients [120]. Hence, one would predict that inhibition of EZH2 expression in T cells would increase the effectiveness of anti-CTLA-4-based immunotherapy, which was indeed observed in this study. In an additional study, the authors explored the anti-tumor action of Dznep, an EZH2 inhibitor, in the setting of nasopharyngeal carcinoma (NPC) in human patients and in mouse models [236]. NPC progression known to be associated with Epstein-Barr Virus-encoded latent membrane protein 1 (LMP1) also drives expression of EZH2 in activated Treg cells, which was antagonized by treatment with Dznep, leading to depletion of Treg cells and enhanced anti-tumor immunity.

Epigenetic drugs can also modulate MDSC function in the TME, leading to improved immunotherapeutic outcomes. In the previous section, we discussed several studies employing a range of HDACi to deplete or inhibit MDSCs, leading to improved antitumor immunity and host response to cancer immunotherapy [117, 118, 142, 143]. Another pathway involved in the epigenetic regulation of intratumoral MDSCs includes H3K27 acetylation by CBP/EP300 bromodomain modulation [237]. In vivo administration of a CBP/EP300-BRD inhibitor (GNE-781) alters intratumoral MDSCs and attenuates established tumor growth in immunocompetent tumor-bearing mice. Mechanistically, inhibition of CBP/EP300-BRD redirects tumor-associated MDSCs from a suppressive to an inflammatory phenotype through downregulation of STAT pathway-related genes and inhibition of Arg1 and iNOS [237].

Tumor-associated macrophages (TAMs) can either inhibit or promote tumor growth depending on their polarization to classically-activated macrophages (M1) or alternatively-activated macrophages (M2). Alpha-ketoglutarate orchestrates macrophage activation to M2 through metabolic and epigenetic reprogramming [219]. As epigenetic mechanisms play significant roles in the polarization, small molecule drugs targeting these epigenetic enzymes have been explored to modulate this polarization [238].

### Activation of natural T cells or CAR T cells through metabolic or/and epigenetic reprogramming

T cell metabolism may be epigenetically-reprogrammed in tumor or other tissue sites [239]. Metabolic pathways can be targeted to enhance T cell-mediated immunity to cancers [223, 240]. As aforementioned, T cells shift to glycolysis to sustain their effector function upon cognate antigen recognition. Restifo, Gattinoni and colleagues showed that by inhibiting glycolytic metabolism one could enhance immunologic memory as well as the anti-tumor function(s) of CD8^+^ T cells [221]. Recently, Zou and collaborators have shown that tumor cells disrupt methionine metabolism in CD8^+^ T cells, leading to lower intracellular levels of methionine and the methyl donor S-adenosylmethionine (SAM), resulting in loss of H3K79me2 in these T cells [241]. This epigenetic change (i.e., loss of H3K79me2) leads to lowered expression of STAT5 and to impaired T cell-mediated immunity, thus uncovering a mechanistic connection between methionine metabolism, histone epigenetic patterns, and T cell immunity in the TME. Moreover, tumor cells outcompete T cells for methionine via a major methionine transporter called SLC43A2. Inhibition of SLC43A2 in tumor cells normalizes methionine metabolism in effector T cells and rescues their functionality, leading to improved spontaneous and ICB-induced anti-tumor immunity in murine tumor models. Thus, targeting cancer methionine signaling may represent an innovative immunotherapeutic strategy.

Many approaches have been developed to promote the epigenetic (re)programming of CD8^+^ T cell differentiation to enhance the effectiveness of immunotherapy [242]. Exhausted T cells undergo metabolic insufficiency with altered signaling cascades and epigenetic profiles, which dampen effector functions and induce poor responsiveness to ICM-targeted therapies [224]. This provides a rationale to develop novel targeted therapeutic strategies.

De novo DNA methylation can promote T cell exhaustion, whereas the inhibition of methylation may promote T cell rejuvenation in vivo. Recently, a group of investigators studied the potential of the DNA demethylation agent decitabine on exhaustion in engineered chimeric antigen receptor T (CAR-T) cells [243]. They found that decitabine augmented CAR-T cell proliferation, production of proinflammatory cytokines and anti-tumor cytolytic functions in vitro and in vivo. Hence, in vitro treatment with decitabine may represent an approach to develop CAR-T cells with improved anti-tumor properties that are capable of mediating enhanced benefits in the clinic.

### Induction of tumor-antigen-specific CD8^+^ T cells

One study addressed the potential of epigenetic modulating agents on the induction of cytotoxic T cell responses against tumor antigens in the setting of malignant pleural mesothelioma [244]. They evaluated the effects of one DNMTi (5-azaCdR) and two HDACi [valproic acid (VPA) and suberoylanilide hydroxamic acid (SAHA)]. Human mesothelioma cells were treated with each epi-drug either alone or in combination. They showed that VPA and SAHA synergized with 5-azaCdR in the killing of MPM cells and coordinately induced increased tumor antigen expression in the remaining viable tumor cells. As a consequence, tumor cells expressing these antigens were then recognized and lysed by antigen-specific CD8^+^ CTLs. In vivo, treatment with the combination of 5-azaCdR and VPA resulted in inhibited tumor growth in association with increased tumor infiltration by immune cells and heightened anti-tumor T cell responses. This provides proof-of-principle for the ability of combination protocols implementing epigenetic modulators to increase tumor antigenicity thereby sensitizing these cells for immune-mediated eradication.

### Cancer cells as non-professional APC: epigenetic enhancement of immunogenicity

It has been long recognized that cancer cells can function as APC even though they have been immunoselected to present tumor antigens poorly as a survival mechanism [245, 246]. Notably, methylation of “immune synapse genes” modulates tumor immunogenicity. Interestingly, when the methylation status of key immune synapse genes was interrogated in cancer cells, a disproportionately high frequency of hypermethylated costimulatory genes and hypomethylation of immune checkpoint genes were observed [247]. Hence, it is not surprising that previous studies suggest that epigenetic modifications may be able to convert a tumor cell that is operationally invisible to the immune system into an effective antigen presenting cell (APC) capable of activating IFN-γ secreting T cells via the MHC class I-presentation pathway.

Multiple approaches have been explored to enhance the immunogenicity and Ag-presenting capacity of tumor cells. HDACs, including Trichostatin A and VPA, have been reported to induce increased expression of TAP, LMP, tapasin genes and MHC class I antigens on melanoma cells and other carcinomas [248, 249]. Both DNMTi and HDACi induce ICD, thus releasing tumor antigens and other danger signals that activate DC and lead to the crosspriming of anti-tumor T cells and may promote Th1-like phenotype [228, 250, 251]. Inhibition of a G9a/DNMT network triggers ICD with a conversion of a cold tumor into a hot tumor, and antitumor immunity [115]. Targeting EZH2, via the use of EZH2 inhibitors GSK126 and EPZ6438, enhanced antigen presentation, antitumor immunity in head and neck cancer [116].

Another way to enhance immunogenicity is by inducing the expression of tumor-specific antigens. DNMT and HDAC inhibitors induce cryptic transcription start site encoded in long terminal repeat [252], and that may generate tumor-specific neoantigens. It is important to note that noncoding regions are the main source of targetable tumor-specific antigens and thus neoantigens [253]. These antigens are induced because of cis- or trans-acting genetic and epigenetic changes in cancer cells. The use of epigenetic modifiers may further increase the expression of these tumor-specific antigens and thus enhance the immunogenicity of cancer cells. Another class of epigenetically inducible antigens are cancer-testis antigens. Weber et al. showed that expression of the MAGE-1 tumor antigen is up-regulated by treating tumor cells with the demethylating agent 5-aza-2′-deoxycytidine [254]. Later, investigators including our own group, showed that many cancer germline antigens such as NY-ESO-1 could be induced by a combination of DNMTi and HDACi in various types of cancer cells [255, 256]. In addition, we showed that tumor cell expression of a homeobox protein, Rhox5, was regulated by epigenetic mechanisms making it a cogent target antigen for vaccines and immunotherapies [257, 258]. Murine modeling has similarly demonstrated that tumor cell expression of cancer/testis antigen P1A is induced by treatment with decitabine, where it functions as a target antigen for adoptively transferred T cells [259].

In addition, as we have discussed earlier, epigenetic reprogramming of tumor cell-intrinsic STING function can augment their antigenicity and presumably their immunogenicity [189].

How key epigenetic mechanisms and epigenetic drugs impact key immune cell populations within the evolving TME and how these changes relate to improved immunotherapy are summarized in a graphic presentation in Fig. 2. Basically, epigenetic drugs, including DNMTi, HDACi, BETi and EZH2i, are believed to work mainly through three pathways or types of cells: 1). they can act on cancer cells to ICD and enhance release/exposure of antigens including neoantigens, to activate DCs and subsequently T cells, with tumor ICD induction and/or enhancing tumor immunogenicity demonstrated for a long list of DNMTi, HDACi and a few HMTi (e.g., EZH2i GSK126 and EPZ6438; G9a/DNMT dual inhibitor CM-272) [114–116, 259]. 2). they may reduce generation and accumulation of MDSCs, while increasing production of ‘good’ chemokines (such as CXCL9, CXCL10) that promote the trafficking of activated (anti-tumor) T cells into tumor tissue as demonstrated for DNMTi, HDACi (MS-275; TSA; SAHA) [117, 118]. 3). they may inhibit Treg cell differentiation and maturation (e.g., EZH2i; BETi: PLX51107) [119–121, 176]. 4). they promote increased production of effector Th1-chemokines and the activation of effector T cells, while synergizing with immune checkpoint blockade for improved therapeutic outcome.

## Clinical trials implementing epigenetic drugs for improved immunotherapy

In the previous sections, we discussed clinical studies using epigenetic modulators to coordinately promote tumor ICD, DC activation, Treg inhibition and the induction of tumor-reactive T cells to effect therapeutic benefit. In this section, we will summarize important findings from completed clinical studies, and then discuss selected examples of ongoing clinical trials.

In the grand scheme of things, multiple epigenetic drugs have been approved for AML, CML, chronic myelodysplastic syndromes, and PTCL after rigorous clinical testing in patients with these diseases (Table 1). However, other than a few types of solid tumors, T-cell lymphoma, and epithelioid sarcoma and refractory follicular lymphoma, to this point, epigenetic drugs have generally failed to demonstrate sufficient efficacy in the advanced disease setting to warrant approval by the FDA or other regulatory authorities.

Yet, a few clinical studies with solid tumors provide reason for hope, as summarized in the following four findings. (1). Tazemetostat, the first-in-class inhibitor, is efficacious in two types of solid tumors. The monotherapy showed clinically meaningful, durable responses and was generally well-tolerated in heavily pretreated patients with relapsed or refractory follicular lymphoma [260], or in advanced epithelioid sarcoma [261]. (2). Combinations of two or more classes of epigenetic drugs may be a good option to enhance treatment anti-tumor efficacy. In one study, the authors demonstrated the combination of azacitidine and romidepsin with IFN-α has a high therapeutic potential based on its targeting of the most aggressive cellular components of colorectal cancer (i.e., metastatic cells and cancer stem cells) and the modulation of key survival and death pathways (including tumor ICD) [230]. In another study, the investigators evaluated combined epigenetic therapy using azacitidine and entinostat (inhibitors of DNA methylation and histone deacetylation, respectively) in extensively pretreated patients with recurrent metastatic non-small cell lung cancer. They showed objective, durable responses in some patients with previously treatment-refractory NSCLC [262]. (3). In preclinical studies, EZH2 inhibitors have been reported to enhance antitumor responses with ICIs (134). In one case report, a patient with SMARCB1-negative chordoma treated with an EZH2 inhibitor (tazemetostat) demonstrated durable, systemic response to radiotherapy for over 2 years. Functional analysis revealed a substantial increase in both intratumoral and stromal infiltration by CD8^+^ cytotoxic T cells and Treg cells, in association with enhanced expression of PD-1 and LAG-3 checkpoint molecules on T cells. These results suggest that EZH2 inhibition promotes sustained antitumor immune responses, leading to immune checkpoint activation [263]. (4). Emerging clinical data also suggest the promise of combination therapies including epi-drugs and ICIs in patients with a range of solid cancers [122].

There are many ongoing clinical studies combining epigenetic drugs and immunomodulatory regimens for the effective treatment of solid cancers. Two recent reviews have compiled lists of ~ 370 ongoing clinical trials using epigenetic modulators for cancer therapy [39, 264]. Readers are directed to Tables in these reviews as valuable resources. In this review, as we are most interested in the combination studies, we have chosen to highlight over a dozen representative clinical trials in which epigenetic drugs are combined with other immunostimulatory agents [265, 266]. These ongoing or just completed phases I/II trials are listed in Table 4. To summarize, these predominantly phase I/II trials have used at least one of the three major classes of specific small molecule inhibitors targeting DNA methylation, histone acetylation or methylation (i.e., DNMTi, HDACi and EZH2i). The other agents employed in the combined regimens are predominantly ICM antagonists. In addition, other immunostimulatory agents have been used, including but not limited to, cancer vaccines, TLR agonists, immunogenic chemotherapeutic agents (e.g, oxaliplatin, olaparib, etc), CAR T cells, or ex vivo expanded TCR T cells, with treatment regimens applied to a variety of tumor types.

**Table 4:** Ongoing clinical trials of epigenetic drugs in combination with immunomodulatory agents for solid cancers (Selected examples)

Identifier	Cancer types and conditions	Epigenetic drug	Other immunostimulatory drug	Trial phase	Estimated enrollment
NCT01928576	Non-small lung cancer	Azacitabine + entinostat or azacitabine alone	Nivolumab (α-PD-1)	II	120
NCT03019003	Head and neck cancer	Azacitidine	Durvalumab + Tremelimumab	Ib/II	59
NCT03024437	Metastatic cancer, renal cancer	Entinostat	Atezolizumab and Bevacizumab	I/II	62
NCT03264404	Pancreatic cancer	Azacitidine	Pembrolizumab (α-PD-1)	II	31
NCT03308396	Advanced kidney cancer, clear cell renal cell carcinoma	Guadecitabine	Durvalumab (α-PD-L1)	Ib/II	59
NCT03576963	Colorectal adenocarcinoma, CpG island methylator phenotype, metastatic microsatellite stable colorectal carcinoma and more	Guadecitabine	Nivolumab	Ib/II	45
NCT04651127	Cervical cancer	Chidamide	Toripalimab (α-PD-1)	Ib/II	40
NCT04562311	Bladder cancer stage IV	Chidamide	Tislelizumab (α-PD-1)	II	43
NCT03829930	Prostate adenocarcinoma	Entinostat	Enzalutamide	I	18
NCT03742245	Relapsed/refractory and/or metastatic breast cancer	Vorinostat	Olaparib	I	28
NCT04553393	Relapsed and/or Refractory B cell Non-Hodgkin’s Lymphoma with huge tumor burden	Chidamide	Decitabine-primed tandem targeting CD19 and CD20 CAR T Cells	I/II	80
NCT04705818	Advanced solid Tumors	Tazemetostat	Durvalumab	II	173

## Future perspectives and challenges

Epigenetic modulators have potential to coordinately impact both tumor cells and non-tumor cells within the TME in a manner that is beneficial to immune recognition of tumor cells in support of improved immune-mediated treatment outcomes. Under the appropriate conditions, these agents can promote tumor ICD, inhibit tumor angiogenesis and hypoxia within the TME, resulting in improved immune cell infiltration and corollary anti-tumor function [266, 267]. Candidate epigenetic biomarkers are anticipated to serve as informative biomarkers for patient stratification, thus maximizing the chance for therapeutic success while minimizing side effects [268]. It is interesting to note that epigenetic mechanisms are often involved in the resistance to chemotherapy, radiation therapy and hormone therapy [269, 270]. Therefore, the application of epigenetic modifiers may re-sensitize the patients with these resistant cancers to conventional therapies. More research needs to be conducted in order to understand the mechanisms by which epigenetic drugs may circumvent therapy resistance in cancer [271].

There remain a number of challenges in using epigenetic modulators as cancer immunotherapeutic agents. The first issue, perhaps the most important issue, is the selectivity of the epigenetic drug. Epigenetic events and their modifying enzymes are ubiquitously distributed across normal and cancer cells. Therefore, it will be critical to determine the most important epigenetic event for targeting that particular type of cancer in an interventional approach. The second issue is that epigenetic therapies have achieved impressive clinical outcomes in hematological malignancies, but not in solid cancers. At this time, investigators continue to empirically explore rational combination treatment strategies in solid cancers to achieve enhanced efficacy. Immunotherapies, especially immune checkpoint blockade (ICB), have achieved striking successes in treating cancer. However, most patients exhibit intrinsic or acquired resistance to ICB-based therapies, necessitating the development of salvage or combination protocols. Targeting epigenetic reprogramming or plasticity represents a new strategy to prevent the emergence of therapeutic resistance to drugs including immunotherapeutic drugs and to enable more consistent clinical responses [271–275]. It is possible that rational combinations incorporating epigenetic modulators may achieve improved objective response rates and durable therapy benefits in patients with advanced solid cancers. In this light, dual hybrids of small molecule inhibitors to exploit multi-targeting of key epigenetic molecules that are dysregulated in cancer may represent particularly promising strategies [276].

## Conclusions

Compelling evidence has demonstrated that epigenetic regulation impacts cancer cells, immune cells, stromal cells, the interactions between cancer cells and immune cells, and the status of the immune TME. As a result, epigenetic modulation by itself can elicit moderate or even robust anti-tumor immunity as an interventional approach. Galluzzi et al. have previously outlined the hallmarks of successful anti-cancer immunotherapies, which include concomitant augmentation of innate and adaptive immunity [277]. Therefore, one rational strategy to further augment immunotherapeutic efficacy is to combine certain epigenetic regulators with one or more classical immunotherapy regimens, such as cancer vaccines, ICIs, oncolytic viruses, CAR-T cells, TCR-T cells, or other novel immunostimulatory agents. Most human solid cancers are immunologically cold tumors and thus hard to treat with immunotherapy [278]. In this case, epigenetic drugs can induce ICD and turn cold tumor hot [114, 115], and they may act in synergy with other immunotherapy regimens. Combination regimens may overcome certain adverse effects and prevent commonly-observed acquired resistance to single-agent immunotherapies. At this time, some combination regimens have shown early promise in clinical studies. In the future, it will be crucial for us to identify the most pivotal epigenetic targets in cancer cells and immune cells for boosting antitumor immunity and developing optimized combination strategies for treating patients with advanced solid cancers.

## Data Availability

The material supporting the conclusion of this review has been included within the article.

## Abbreviations

AML: Acute myeloid leukemia
APC: Antigen presenting cells
CAR-T cells: Chimeric antigen receptor T cells
CTCL: Cutaneous T cell lymphoma
Epi-drugs: Epigenetic drug
EZH2: Enhancer of zeste homolog 2
DAMPs: Danger-associated molecular patterns
DC: Dendritic cells
DNMT: DNA methyltransferase
DNMTi: DNA methyltransferase inhibitor
HAT: Histone acetyltransferase
HIF: Hypoxia-induced transcription factor
HDAC: Histone deacetylase
HDACi: Histone deacetylase inhibitor
HDM: Histone demethylase
HMT: Histone methyltransferase
ICD: Immunogenic cell death
ICM: Immune checkpoint molecule
ICI: Immune checkpoint inhibitor
MDSC: Myeloid-derived suppressor cells
5mC: 5-methylcytosine
NSCLC: Non-small cell lung cancer
PAMPs: Pathogen-associated molecular patterns
PD-1: Programmed death protein-1
PD-L1: Programmed death ligand-1
PRMT: Protein arginine methyltransferase
PTCL: Peripheral T-cell lymphoma
HKMT: Histone lysine methyltransferase
TAM: Tumor-associated macrophages
TET: Ten-eleven translocation (family enzymes)
TGF-β: Transforming growth factor-β
TME: Tumor microenvironment
Treg: Regulatory T cells
